# Analysis of phase noise effects in a coupled Mach–Zehnder interferometer for a much stabilized free-space optical link

**DOI:** 10.1038/s41598-021-81522-y

**Published:** 2021-01-21

**Authors:** Byoung S. Ham

**Affiliations:** Center for Photon Information Processing, School of Electrical Engineering and Computer Science, Gwangju Institute of Science and Technology, 123 Chumdangwagi-ro, Buk-gu, Gwangju, 61005 South Korea

**Keywords:** Physics, Quantum optics

## Abstract

Recently, new physics for unconditional security in a classical key distribution (USCKD) has been proposed and demonstrated in a frame of a double Mach–Zehnder interferometer (MZI) as a proof of principle, where the unconditional security is rooted in MZI channel superposition. Due to environmental phase noise caused by temperature variations, atmospheric turbulences, and mechanical vibrations, free-space optical links have been severely challenged for both classical and quantum communications. Here, the double MZI scheme of USCKD is analyzed for greatly subdued environment-caused phase noise via double unitary transformation, resulting in potential applications of free-space optical links, where the free-space optical link has been a major research area from fundamental physics of atomic clock and quantum key distribution to potential applications of geodesy, navigation, and MIMO technologies in mobile communications systems.

## Introduction

In quantum key distribution (QKD) technologies^1–15^, unconditional security is provided by the no-cloning theorem of quantum mechanics using canonical variables, resulting from randomness via the Heisenberg uncertainty principle^16^. Randomness is the fundamental basis of unconditional security according to information theory^17^. In practice, however, quantum loopholes such as imperfect detectors and lossy quantum channels are major obstacles, resulting in conditional security as in classical technologies of algorithm-based protocols^9–15^. In addition to quantum loopholes, development of deterministic single photons or entangled photon-pair generators is far behind commercial implementations. Moreover, the deployment of long-distance quantum key distributions seems to not be possible due simply to the non existence of quantum repeaters^18^. In addition, the QKD key rate is extremely low compared with classical counterparts, and QKDs are not compatible with any classical systems in the real world such as fiber backbone networks and wireless mobile networks. Thus, implementations of QKD for practical applications may not be plausible in the near future. In other words, unconditionally secured information communications in the real world such as in nationwide on-line banking via quantum internet^19^ are not possible with current QKD systems.

Since the recent investigation of quantumness regarding anticorrelation and photon bunching on a beam splitter (BS)^20–27^, a completely different physics for nonclassicality has been discussed^28–31^ and experimentally demonstrated^32,33^ for a coherence version of photonic de Broglie waves (CBW) and the unconditional security of classical key distributions (USCKD), where USCKD and CBW share the same physics as heads and tails of a coin^31^. This new finding of coherence quantumness is macroscopic based on bright coherent light with quantum superposition of two Mach–Zehnder interferometers via a specific coupling method. So far, quantum information has been limited to the microscopic world composed of a few atoms or photons, relying on the particle nature of duality^34^. According to the new understanding of quantumness on a BS, photon bunching or anticorrelation, however, is based on the wave nature of photons, resulting in coherence quantum information, where such nonclassical features of anticorrelation cannot be obtained classically. For example, the physics of CBW lies in the controllable higher-order quantum superposition among independent Mach–Zehnder interferometers (MZIs)^27,32^, where the coupling method plays a key role. Like a coupled two-mode pendulum model in classical physics^35^, the quantumness in CBW has also been investigated using a tensor product of independent phase bases in coupled MZIs, where the phase bases satisfy the orthonormal conditions of a Hilbert space^31^.

The heart of unconditional security in both QKD and USCKD is in the randomness of eavesdropping for the measurements of distributed keys. In QKD, the randomness is in Heisenberg’s uncertainty principle of conjugate variables, where each variable set is composed of two orthogonal bases and the no-cloning theorem provides the bottom line of no copying. On the other hand, the eavesdropping randomness of orthogonal bases in USCKD is sought from the path superposition of MZI, where the realization of randomness is provided by key distribution determinacy between two remote parties via double unitary transformations^28,33^. Importantly, USCKD has nothing to do with no-cloning theorem of QKD based on single photons or entangled photon pairs, where the key carrier of USCKD is conventional laser light such as in fiber-optic communications system. Regarding USCKD, however, the two-channel layout of transmission lines should be vulnerable to environment-caused phase noises induced by temperature variations, mechanical vibrations, and atmospheric turbulences. It is well known that an active phase control is necessary for the applications based on MZI^36–39^. Although state-of-the-art laser locking technologies are well implemented for such applications, the active control is still challenging and limits the maximum performance of the system. Here, we investigate greatly subdued phase noise characteristics of USCKD in the double Mach–Zehnder interferometer, where the phase relaxed characteristics can be applied for various optical links of wireless (free-space) communications systems for farther transmission distance, otherwise limited by a few kilometers.

## Results

**Figure 1:**
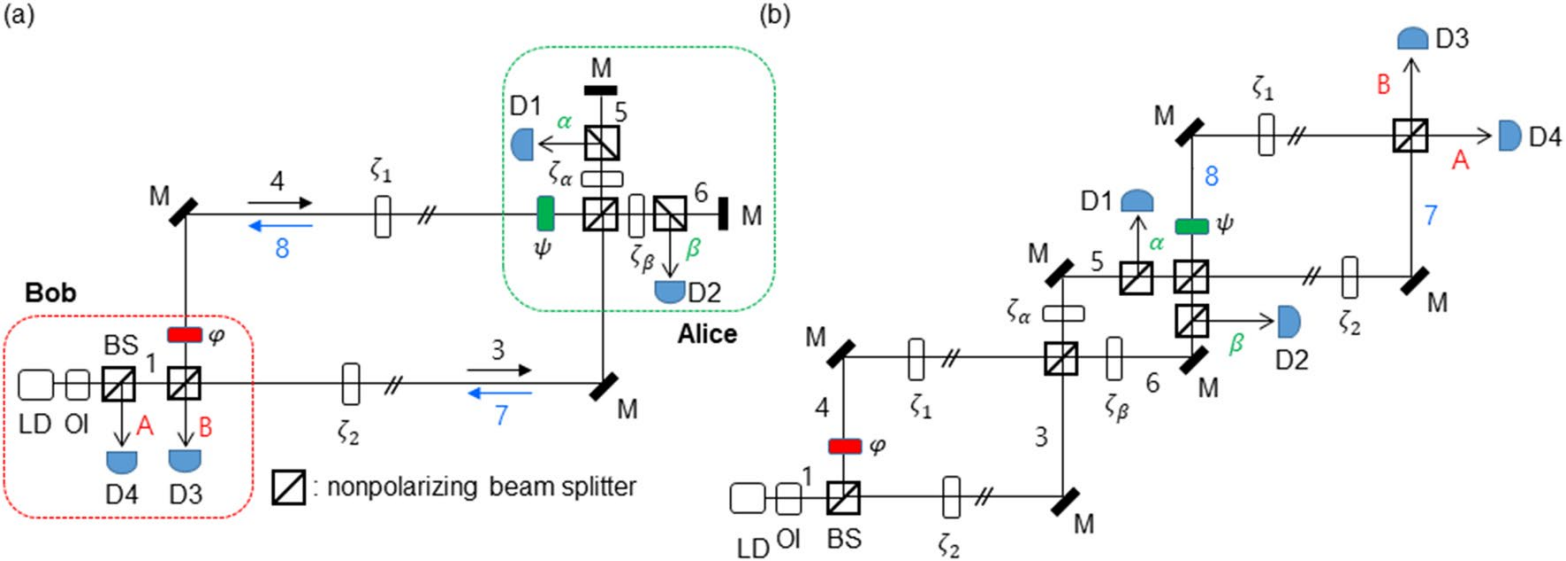
A schematic of USCKD. (**a**) An original schematic of USCKD. (**b**) An unfolded schematic of (**a**). LD: laser diode, OI: optical isolator, BS: nonpolarizing beam splitter, D: detector, M: mirror. ${\zeta }_{j}$: environmental phase noise. The numbers indicate corresponding electric fields.

Figure 1 shows a schematic of the USCKD, where the environment-caused phase noises incurred in transmission channels of MZI are perfectly and automatically compensated via a round-trip transmission scheme. In Fig. 1(a), the shared transmission channels of MZI between two remoted parties, Alice and Bob, are not quantum but classical, where Bob controls the phase shifter $\mathrm{\varphi }$ and detectors D3 and D4, while Alice controls the phase shifter $\uppsi$ and detectors D1 and D2. Here, ‘classical’ means that an eavesdropper can copy the light carrier in channels without revealing the eavesdropper’s existence to both Alice and Bob, as allowed in classical cryptographic systems. The ${\zeta }_{j}$ represents environment-caused phase noise in each transmission channel, where ${\zeta }_{1}\ne {\zeta }_{2}$ due to channel independency. The phase $\uppsi$ is for the light returned by Alice, where $\uppsi$ is invisible to the outbound lights ‘3’ and ‘4’.

According to USCKD^30^, Bob prepares a key with a random phase basis, $\mathrm{\varphi }\in \left\{0,\pi \right\}$, where the phase bases are orthonormal to each other in the MZI system. Alice confirms Bob’s prepared key with her phase basis choice, $\uppsi \in \left\{0,\pi \right\}$. Here, Alice’s confirmation depends on a protocol, whether the confirmation can be limited to the same bases or all bases (see the Supplementary Information of ref.^30^). If the bases are the same each other, the double unitary transformation results in an identity relation, otherwise an inversion relation for different basis combinations^30^. This two-channel key distribution process of USCKD is perfectly deterministic due to MZI directionality: If Bob’s basis choice is for $\mathrm{\varphi }=\uppi$, Alice’s detector D1 must click; If Bob’s choice is $\mathrm{\varphi }=0$ and Alice’s choice is $\uppsi =0$, Bob’s detector D4 clicks. The unconditional security is rooted in the superposition-caused measurement randomness in the shared MZI channels^30^. A sophisticated Eve, of course, can use the same measurement tool as Bob’s or Alice’s. However, the information extraction chance by Eve is 50% on average due to the indistinguishability in the superposed channels of MZI, resulting in measurement randomness. To avoid classical attacks such as memory-based attacks, an authentication or network initialization process is required^30^.

Using matrix representations, the following analytic derivations are obtained for Fig. 1:

1 $$\left[\begin{array}{c}{E}_{\alpha }\\ {E}_{\beta }\end{array}\right]={\left[MZI\right]}_{1}\left[\begin{array}{c}{E}_{0}\\ 0\end{array}\right]=\frac{1}{2}{e}^{i{\zeta }_{2}}\left[\begin{array}{cc}1-{e}^{i{\varphi }^{\mathrm{^{\prime}}}}& i\left(1+{e}^{i{\varphi }^{\mathrm{^{\prime}}}}\right)\\ i\left(1+{e}^{i{\varphi }^{\mathrm{^{\prime}}}}\right)& -\left(1-{e}^{i{\varphi }^{\mathrm{^{\prime}}}}\right)\end{array}\right]\left[\begin{array}{c}{E}_{0}\\ 0\end{array}\right],$$

where ${\left[MZI\right]}_{1}=\left[BS\right]\left[\varphi \right]\left[\zeta \right]\left[BS\right]$, $$\left[BS\right]=\frac{1}{\sqrt{2}}\left[\begin{array}{cc}1& i\\ i& 1\end{array}\right]$$, $$\left[\varphi \right]=\left[\begin{array}{cc}1& 0\\ 0& {e}^{i\varphi }\end{array}\right]$$, $\upzeta ={\zeta }_{2}-{\zeta }_{1}$, and ${\varphi }^{{\prime}}=\varphi -\zeta$. Thus, the effective channel noise $\upzeta$ due to the environmental phase fluctuations results in:

2 $${I}_{\alpha }=\frac{1}{2}\left[1-\mathrm{cos}(\varphi -\zeta )\right]{I}_{0},$$

3 $${I}_{\beta }=\frac{1}{2}\left[1+\mathrm{cos}\left(\varphi -\zeta \right)\right]{I}_{0}{I}_{0}.$$

Thus, the output intensities of ${I}_{\alpha }$ and ${I}_{\beta }$ for a fixed $\mathrm{\varphi }$ are time dependent, whose average is bounded by the classical limit of ${I}_{0}/2$. This means that the control of environmental phase fluctuations is critical in MZI interferometry^36–39^.

For the returned light to Bob’s side by Alice in Fig. 1, the matrix representations are as follows (see Section A of the Supplementary Materials):

4 $$\left[\begin{array}{c}{E}_{A}\\ {E}_{B}\end{array}\right]={\left[MZI\right]}_{2}\left[{\zeta }_{\alpha \beta }\right]\left[\begin{array}{c}{E}_{\alpha }\\ {E}_{\beta }\end{array}\right]=\frac{1}{4}{e}^{i\left({{\zeta }_{2}+\zeta }_{2}^{{\prime}}+{\zeta }_{\beta }\right)}\left[\begin{array}{cc}-\left(1-{e}^{i{\zeta }^{{{\prime}}{{\prime}}}}\right)\left(1+{e}^{i\left({{\varphi }^{{\prime}}+\psi }^{{\prime}}\right)}\right)-\left(1+{e}^{i{\zeta }^{{{\prime}}{{\prime}}}}\right)\left({e}^{i{\varphi }^{{\prime}}}+{e}^{i{\psi }^{{\prime}}}\right)& -i\left[\left(1-{e}^{i{\zeta }^{{{\prime}}{{\prime}}}}\right)\left(1-{e}^{i\left({{\varphi }^{{\prime}}+\psi }^{{\prime}}\right)}\right)+\left(1+{e}^{i{\zeta }^{{{\prime}}{{\prime}}}}\right)\left({e}^{i{\psi }^{{\prime}}}-{e}^{i{\varphi }^{{\prime}}}\right)\right]\\ -i\left[\left(1-{e}^{i{\zeta }^{{{\prime}}{{\prime}}}}\right)\left(1-{e}^{i\left({{\varphi }^{{\prime}}+\psi }^{{\prime}}\right)}\right)+\left(1+{e}^{i{\zeta }^{{{\prime}}{{\prime}}}}\right)\left({e}^{i{\psi }^{{\prime}}}-{e}^{i{\varphi }^{{\prime}}}\right)\right]& \left(1-{e}^{i{\zeta }^{{{\prime}}{{\prime}}}}\right)\left(1+{e}^{i\left({{\varphi }^{{\prime}}+\psi }^{{\prime}}\right)}\right)-\left(1+{e}^{i{\zeta }^{{{\prime}}{{\prime}}}}\right)\left({e}^{i{\varphi }^{{\prime}}}+{e}^{i{\psi }^{{\prime}}}\right)\end{array}\right]\left[\begin{array}{c}{E}_{0}\\ 0\end{array}\right]$$

where ${\left[MZI\right]}_{2}=\left[BS\right]\left[\psi \right]\left[\zeta \right]\left[BS\right]$, $$\left[\psi \right]=\left[\begin{array}{cc}1& 0\\ 0& {e}^{i\psi }\end{array}\right]$$, $$\left[{\zeta }_{\alpha \beta }\right]=\left[\begin{array}{cc}{e}^{i{\zeta }_{\alpha }}& 0\\ 0& {e}^{i{\zeta }_{\beta }}\end{array}\right]$$, ${\upzeta }^{{\prime}}={\upzeta }_{2}^{{\prime}}-{\upzeta }_{1}^{{\prime}}$, ${\zeta }^{{{\prime}}{{\prime}}}={\zeta }_{\alpha }-{\zeta }_{\beta }$, and ${\psi }^{{\prime}}=\psi -{\zeta }^{{\prime}}$. Here $\left[{\zeta }_{\alpha \beta }\right]$ is the environment-caused phase noise occurred in the coupling MZI between two main MZIs of ${\left[MZI\right]}_{1}$ and ${\left[MZI\right]}_{2}$, and ${\upzeta }^{{\prime}}$ is the phase noise difference between two channels in ${\left[MZI\right]}_{2}$. Although ${\upzeta }^{{\prime}}$ is different from $\zeta$ in general, they can be treated to be equal in a short traveling distance or slowly varying noise. In this case, the transit time between the outbound and inbound light fields should be much shorter than that of atmospheric turbulence-time causing a π phase shift. Based on the atmospheric turbulence rate is ~ kHz, the transmission distance satisfying $\zeta \sim {\upzeta }^{{\prime}}$ condition should be a few tens of km^40^. Here, the relative noise variation $\upzeta$ and ${\zeta }^{{\prime}}$ is expected to be much slower than the individual channel noise ${\zeta }_{j}$ and ${\zeta }_{j}^{{\prime}}$.

(i) For $${\zeta }^{{{\prime}}{{\prime}}}=0$$ and $$\zeta ={\upzeta }^{{\prime}}$$,

For ${\zeta }^{{{\prime}}{{\prime}}}=0$, Eq. (4) is represented as:

5 $$\left[ {\begin{array}{*{20}c} {E_{A} } \\ {E_{B} } \\ \end{array} } \right] = \frac{ - 1}{2}e^{{i\left( {\zeta_{1} + \zeta_{2} + \zeta_{\beta } - \varphi } \right)}} \left[ {\begin{array}{*{20}c} {\left( {e^{{i\left( {\psi - \varphi } \right)}} + 1} \right)} & {i\left( {e^{{i\left( {\psi - \varphi } \right)}} - 1} \right)} \\ {i\left( {e^{{i\left( {\psi - \varphi } \right)}} - 1} \right)} & {\left( {e^{{i\left( {\psi - \varphi } \right)}} + 1} \right)} \\ \end{array} } \right]\left[ {\begin{array}{*{20}c} {E_{0} } \\ 0 \\ \end{array} } \right].$$

Thus, the output intensities for Eq. (5) are as follows:

6 $$I_{A} = \frac{1}{4}\left[ {1 + e^{{i\left( {\psi - \varphi } \right)}} } \right]\left[ {1 + e^{{ - i\left( {\psi - \varphi } \right)}} } \right]I_{0} = \frac{1}{2}\left[ {1 + {\text{cos}}\left( {\psi - \varphi } \right)} \right]I_{0} .$$

7 $$I_{B} = \frac{1}{4}\left[ {1 - e^{{i\left( {\psi - \varphi } \right)}} } \right]\left[ {1 - e^{{ - i\left( {\psi - \varphi } \right)}} } \right]I_{0} = \frac{1}{2}\left[ {1 - {\text{cos}}\left( {\psi - \varphi } \right)} \right]I_{0.}$$

**Figure 2:**
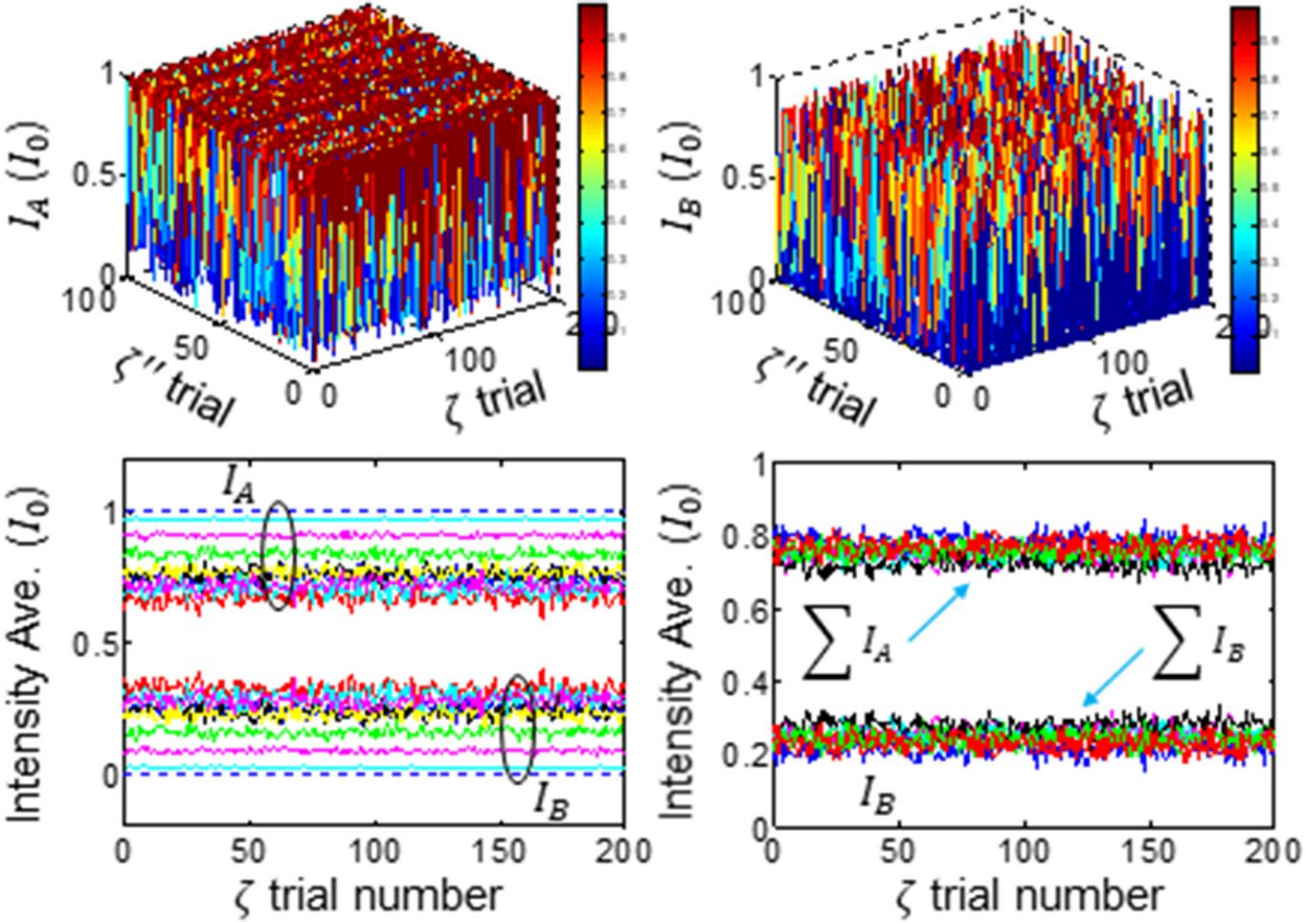
Numerical calculations of Eq. (4) for ${\zeta }^{{{\prime}}{{\prime}}}\ne 0$ and $\mathrm{\varphi }=\uppsi =0$. At each trial random phase is applied, where the r range of ${\zeta }^{{{\prime}}{{\prime}}}$ varies for a fixed range of $\upzeta$. Upper panel: Individual output intensities for random phase variations of ${\zeta }^{{{\prime}}{{\prime}}} (2\uppi )$ and $\upzeta (2\uppi )$. Lower panel (left): Intensity average over ${\zeta }^{{{\prime}}{{\prime}}}$, whose random phase variation is $0(dotted);\frac{\pi }{4}(cyan);\frac{\pi }{2}(magenta);\frac{3\pi }{4}(green);\pi (blue);\frac{5\pi }{4}(red);\frac{3\pi }{2}(black/yellow);\frac{7\pi }{4}(green);2\pi (red$). Lower panel (right): 10 averaged intensities for ${\zeta }^{{{\prime}}{{\prime}}}=2\pi$. $\Delta\upzeta =\left|\zeta -{\upzeta }^{{\prime}}\right|=0$.

As a result, Fig. 1 satisfies the identity and inversion relations of the original protocol of USCKD with no influence of environmental phase noises if ${\zeta }^{{{\prime}}{{\prime}}}=0$ and $\zeta ={\upzeta }^{{\prime}}$ for a short distance. This noise-free result, hower, may be difficult to be applied for conventional single channel-based communication systems, because the condition of $\zeta ={\upzeta }^{{\prime}}$ does not mean that each channel is noise free but the relative noise between MZIs does. Here, the relative noise between two transmission channels such as ‘3’ and ‘4’ can be much weaker compared with each channel noise (see “Discussion”).

(ii) For ${\zeta }^{{{\prime}}{{\prime}}}\ne 0$ and $\zeta ={\upzeta }^{{\prime}}$,

If the intermediate coupling MZI is exposed to environmental phase noises freely, then the output fields of Eq. (4) are affected by both ${\upzeta }^{{{\prime}}{{\prime}}}$ and $\upzeta$. The related numerical calculations are shown in Fig. 2 as both functions of ${\zeta }^{{{\prime}}{{\prime}}}$ and $\upzeta$ for $\mathrm{\varphi }=\uppsi$ (see the Section B in the Supplementary Information for $\mathrm{\varphi }\ne\uppsi$). Here, the phase noise of ${\zeta }^{{{\prime}}{{\prime}}}$ and $\upzeta$ represents random noise range. The phase noise difference between the channel MZIs is set to be equal, i.e., $\zeta ={\upzeta }^{{\prime}}$, even though $\zeta \ne 0$ and ${\zeta }^{{\prime}}\ne 0$, satisfying slowly varying noise or a short transmission distance.

For the numerical calculations in Fig. 2, the output field intensities are calculated for both randomly varying ${\zeta }^{{{\prime}}{{\prime}}}$ and $\upzeta$. In the upper panels, the output intensities of ${I}_{A}$ and ${I}_{B}$ result in random fluctuations between 0 and 1 in the unit of the input field intensity ${I}_{0}$ due to the random phase noise between 0 and $2\uppi$. To analyze the results, output intensities averaged for all ${\zeta }^{{{\prime}}{{\prime}}}$ ($\upzeta$) values at each random phase noise of $\upzeta$ (${\zeta }^{{{\prime}}{{\prime}}}$) are represented in the lower panels, where the analytical results of Eqs. (6) and (7) are shown by the dotted lines (see the lower left panel). The output fields ${I}_{A}$ and ${I}_{B}$ are effective for the invariance characteristics to the random channel noises of ${\zeta }_{1}$ and ${\zeta }_{2}$ if ${\zeta }^{{{\prime}}{{\prime}}}=0$ and $\zeta ={\upzeta }^{{\prime}}$ with more than 50% visibility. Starting from this reference value, the average value of the output intensity ${I}_{A}$ (${I}_{B}$) gradually reduces (increases) from 1 (0) to 0.75 (0.25) as the random phase noise range of ${\zeta }^{{{\prime}}{{\prime}}}$ increases to $\uppi$. If the random phase variation range of ${\zeta }^{{{\prime}}{{\prime}}}$ increases further toward $2\uppi$, each average output intensity fluctuates up and down in a small range across 0.75 and 0.25, respectively.

**Figure 3:**
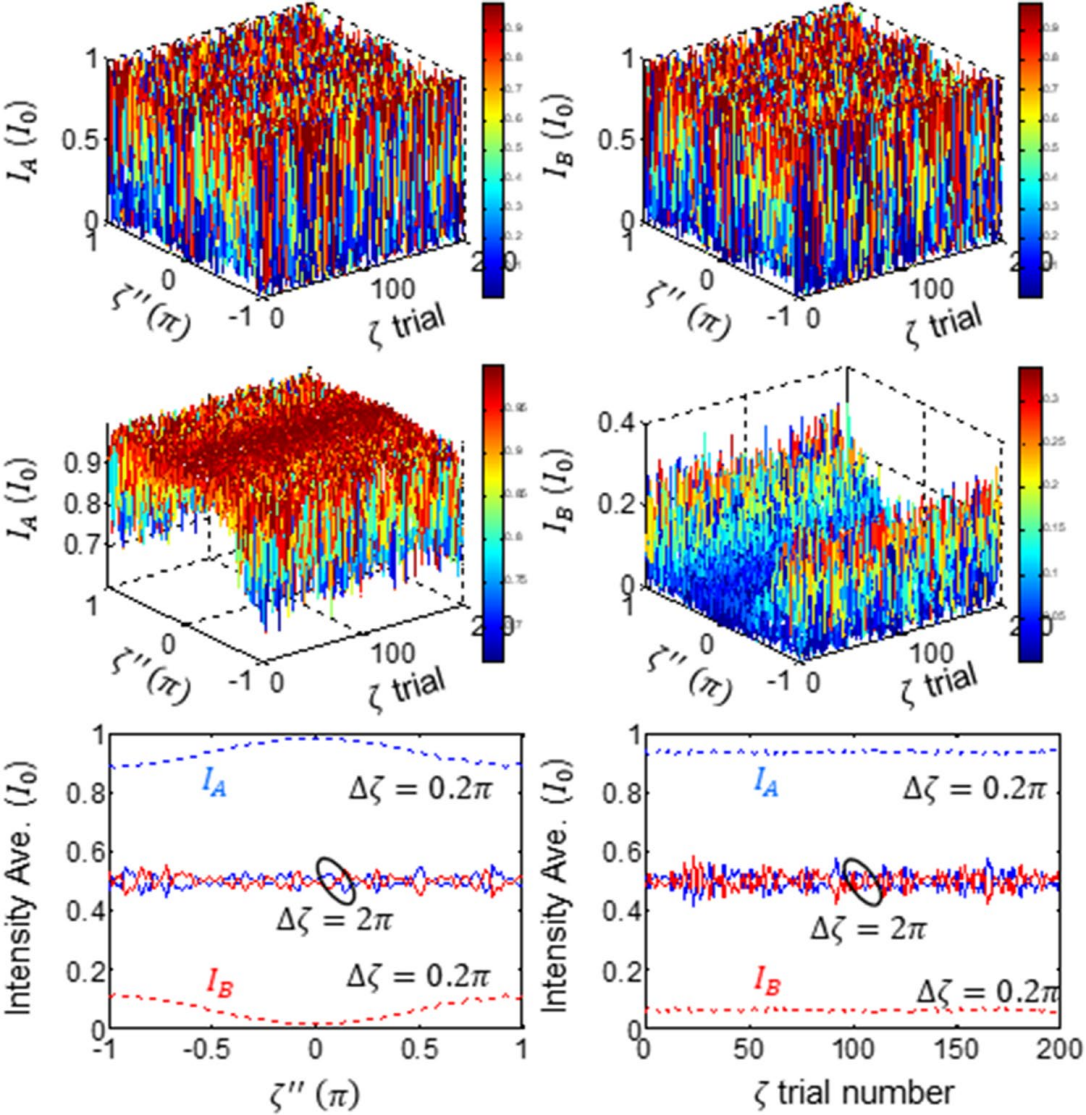
Numerical calculations for the output fields as a function of ${\zeta }^{{{\prime}}{{\prime}}}$ for $\zeta \ne {\upzeta }^{{\prime}}$. (Upper panel) $\mathrm{\Delta \zeta }=2\uppi$. (Middle panel) $\mathrm{\Delta \zeta }=0.2\uppi$. (lower panel) dotted curves: $\mathrm{\Delta \zeta }=0.2\uppi$, solid curves: $\mathrm{\Delta \zeta }=2\uppi$. $\mathrm{\Delta \zeta }\equiv \left|\zeta -{\upzeta }^{{\prime}}\right|$.

The lower right panel shows ten repeated data for a maximum random phase range of ${\zeta }^{{{\prime}}{{\prime}}}=2\pi$, where each intensity is clearly and completely separated even under the random phase noises of both ${\zeta }^{{{\prime}}{{\prime}}}$ and $\upzeta$. Because both intensity would vary between 0 and 1, the lowest (highest) average value of ${I}_{A}$ (${I}_{B}$) should be ${I}_{0}/2$ in the average. As a result, the average value of the output intensities for all possible ${\zeta }^{{{\prime}}{{\prime}}}$ are expected to be a half of each maxima, resulting in ${3I}_{0}/4$ (${I}_{0}/4$) for ${I}_{A}$ (${I}_{B}$). Thus, USCKD is robust for the key distribution even under full scale of phase noise if $\zeta ={\upzeta }^{{\prime}}$, where the detection bound for ${I}_{A}$ (${I}_{B}$) can be set lower (higher) than for the key distribution. This is the first significance in the environment-caused noise immune characteristic of USCKD.

(iii) For $${\zeta }^{{{\prime}}{{\prime}}}\ne 0$$ & $$\zeta \ne {\upzeta }^{{\prime}}$$,

For a general scheme of USCKD with $\zeta \ne {\upzeta }^{{\prime}}$ between outbound (‘3’ and ‘4’) and inbound (‘7’ and ‘8’) lights, respectively, Eq. (4) is numerically calculated in Fig. 3. For this, the control MZI noise varies linearly, while the transmission channel noise difference at each MZI varies randomly. The upper panels of Fig. 3 show output intensities fluctuating in a full range for $\Delta\upzeta \equiv \left|\zeta -{\upzeta }^{{\prime}}\right|=2\pi$ as expected.

In the middle panels of Fig. 3, the relative transmission channel noise range is reduced to $\Delta\upzeta =0.2\pi$. Unlike the upper panels, output intensity fluctuations are greatly reduced, whose visibility is bigger than 40% even for ${\zeta }^{{{\prime}}{{\prime}}}=2\pi$. Especially for the phase-locked case with ${\zeta }^{{{\prime}}{{\prime}}}\sim 0$, individual output intensity fluctuations are greatly suppressed. As shown in the dotted curve in the lower left panel, the average output intensities are also greatly stabilized to be nearly immune to the noise, regardless of ${\zeta }^{{{\prime}}{{\prime}}}$. As $\Delta\upzeta$ increases, the output intensity fluctuation also increases and finally reaches at the maximum when $\Delta\upzeta =2\uppi$ (see the blue and red solid curves in the middle).

The lower right panel of Fig. 3 shows the average output field intensities over ${\zeta }^{{{\prime}}{{\prime}}}$ with respect to the transmission channel noise difference $\Delta\upzeta$. In general, the intermediate MZI is easy to control, while the transmission channel is not. The present USCKD scheme of double MZI channels makes the system robust to the environment-caused phase noise if $\Delta\upzeta \ll\upzeta , {\zeta }^{{\prime}}$ (see the dotted lines). Even without active phase control necessary to all optical links^41–45^, thus, the double MZI scheme of USCKD can support channel noise-immune communication protocol, where the upper bound of transmission distance is determined by the relative phase noise $\Delta\upzeta$ between two MZIs within the transit time. Because the atmospheric turbulence is much smaller and slower than the individual channel noise ${\upzeta }_{j}\sim {\zeta }_{j}^{{\prime}}$, this is the second significance of USCKD for the phase noise reduction.

**Figure 4:**
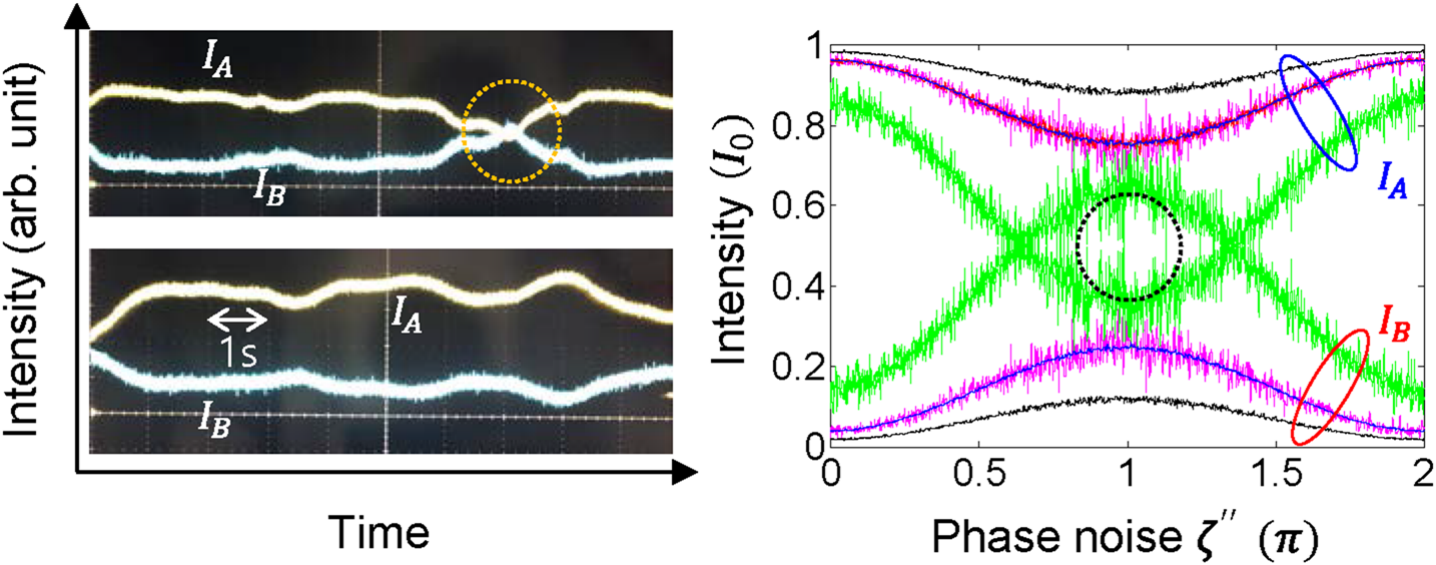
Experimental demonstrations of Eq. (4) for ${\zeta }^{{{\prime}}{{\prime}}}\ne 0$. (Left) Experimental results for the average of n = 10 (see “Methods”). (Right) Numerical analysis for random phase noise variation of ${{\zeta^{\prime\prime}}}$ with averaging for $\Delta {\upzeta } = {\uppi }/30$ (black), ${\uppi }/20$ (magenta/blue), and ${\uppi }/10$ (green). Average n = 500 (black and blue); n = 50 (cyan and magenta); n = 5 (green).

Figure 4 shows experimental data and its analysis. The left panel of Fig. 4 is experimental results related with the lower panels of Fig. 3, where both ${\zeta }^{{{\prime}}{{\prime}}}$ and $\zeta$ affect the output fields under a slow phase noise variation. The fairly noise-subdued USCKD has been reported in ref. 33 for the same setup without phase control, where the experimental setup is simply placed in a quiet, calm, closed room. In the left panel of Fig. 4, the output fields are average 10 times internally by a Tektronix digital oscilloscope. Under typical lab conditions, the output fields slowly fluctuate and cross over the half line (see the dotted circle). This slight cross-over becomes severe as $\Delta\upzeta$ increases by atmospheric turbulence.

In the right panel of Fig. 4, the slight crossover observed in the experiments is analyzed with different $\mathrm{\Delta \zeta }$ over the random $\upzeta -$ caused intensity values for a specific noise range of ${\zeta }^{{{\prime}}{{\prime}}}$. For this, ${\zeta }^{{{\prime}}{{\prime}}}$ linearly varies from 0 to $2\uppi$. As analyzed in Eqs. (4)–(7), the output intensities are nearly immune to phase noise if ${\zeta }^{{{\prime}}{{\prime}}}=0$, as is numerically confirmed at both ends. For ${\zeta }^{{{\prime}}{{\prime}}}\ne 0$, the random phase noise results in increased noise effect, where this intensity noise increases as $\Delta\upzeta$ increases as represented by colored curves. As shown in the magenta with blue curve for the same $\Delta\upzeta =\uppi /20$, higher averaging results in less fluctuation as expected. Thus, the crossover in the experiment is well explained by both $\Delta\upzeta$ and ${\zeta }^{{{\prime}}{{\prime}}}$. Because ${\zeta }^{{{\prime}}{{\prime}}}$ can be set to be nearly zero with an active phase control, the $\Delta \zeta$ controllability is much more relaxed (see the well separated bundles at ${\zeta }^{{{\prime}}{{\prime}}}=0$). For a short transmission distance, the USCKD is promising for phase noise-immune free-space communications due to much less variation in the difference phase $\Delta \zeta$ between two MZIs rather than individual phase noise difference $\upzeta$ and ${\zeta }^{{\prime}}$.

## Discussion

Unlike the one-way MZI system of Eqs. (1)–(3)^38,39^, Eq. (4) is for the round-trip MZI system of USCKD, representing the environmental phase-noise effect in Fig. 1. As analyzed in Eqs. (5)–(7), the transmission channels of the shared MZI are inherently sensitive to environmental noise such as atmospheric turbulences. Because intermediate coupling MZI can be controlled to be ${\zeta }^{{{\prime}}{{\prime}}}=0$ by the state-of-the-art laser locking technologies, the output intensity noise fluctuations of the USCKD become fairly subdued if the environmental phase noise for the transmission MZI is controlled less than $\uppi /10$. This less environmental phase noise effect to the present USCKD system gives a great benefit for free-space communications, otherwise atmospheric turbulence is the critical obstacle for optical links in both quantum^41–43^ and classical^44,45^ communications.

Detailed studies for an optimum transmission distance by the proposed noise-immune USCKD have not been done yet. However, it is the common interest in the area of optical links for geodesy, navigation, etc. Recently 1 km distance QKD through MZI channels have been demonstrated using active phase locking system^39^. This is a typical phase control for ${\upzeta }$ or ${{\zeta^{\prime}}}$ in Eq. (4) as usual. The relative phase control between inbound and outbound MZIs are a much easier task because it is a matter of ${{\Delta \zeta }} = \left| {\zeta - \zeta^{\prime}} \right|$, in which ${{\Delta \zeta }}$ is a function of the first-order derivative in ${\upzeta }$ and $\zeta^{\prime}$: $\frac{{d\left( {{\Delta }\zeta } \right)}}{dt} = \frac{{d\varphi_{1} }}{dt} - \frac{{d\varphi_{2} }}{dt}$. If two MZI channels are deployed in a space very near each other, then $\frac{{d\varphi_{1} }}{dt}\sim \frac{{d\varphi_{2} }}{dt}$, resulting in ${{\Delta \zeta }}\sim 0$. Based on the data from the optical link, the atmospheric turbulence has linear relation between stability and accumulation time^44^. Considering a successful demonstrations of a few tens of km optical link, the present method of USCKD can be applied for the same optical link with much farther distance due to ${{\Delta \zeta }}\sim 0$.

## Conclusion

We analyzed, discussed, and experimentally demonstrated for the environmental phase noise effect on USCKD protocol, where the phase noise control is critical to practical usage especially for free-space communications. The noise effect on USCKD has been analyzed by noise parameters, where the intermediate coupling MZI can be well controlled by using the state-of-the-art laser locking technologies. Based on this phase locking, the transmission channel noise control plays a major role for the system performance, where the output fields fluctuation is well subdued if the transmission channel noise difference between inbound and outbound channels is controlled less than ${\uppi }/10$. Because the difference phase noise between MZIs is much more stable than individual channel noises, the round-trip MZI transmission scheme of USCKD gives a great benefit to the practical applications of free-space optical links. Although perfect noise immune USCKD cannot be reached with zero phase noise in all MZIs of USCKD, a short transmission distance for the key distribution may allow phase noise-immune characteristics even without tight active phase locking. Thus, the present studies may open the door to a new realm of secured free-space information communications.

## Methods

Numerical calculations: In Figs. 2, 3, 4, home-made Matlab programs are used for Eq. (4) and numerically calculated, where the random phase noise is obtained from the rand(1) commend. In Figs. 2, 3, 4, various phase noise range for $\zeta^{\prime\prime}$ and ${\upzeta }$ was controlled by multiplying a certain number to the output of rand(1).

Experiments: In Fig. 4, the wavelength of the input light was 606 nm from Toptica TA-SHG pro. The optical power of the input light was ~ 1 mW. After splitting by the first beam splitter in Fig. 1, each channel was controlled by each AOM driven by synchronized rf generators (PTS 160/250; Tektronix AFG3102), resulting in the same initial phase (see ref. 29 for details). The data was captured in the screen of an oscilloscope (Tektronix DPO 5204B) via avalanche photodiodes (Hamamatsu C12703). The experimental layout is for Fig. 1(b), where the transmission length of each MZI is ~ 70 cm, and independent (random) phase noise occurs in both MZIs, satisfying non-zero phase noise.

## Supplementary Information

Supplementary Information.
