# Supplementary material for: Analysis of phase noise effects in a coupled Mach–Zehnder interferometer for a much stabilized free-space optical link
Source: Sci Rep. 2021 Jan 21;11:1900. doi: 10.1038/s41598-021-81522-y (PMC7820431; doi:10.1038/s41598-021-81522-y)
Supplement: Supplementary file 1 — Supplementary Information. [file 41598_2021_81522_MOESM1_ESM.pdf]

Supplementary Information for

"Analysis of phase noise effects in a coupled Mach-Zehnder interferometer for a much stabilized free-space optical link" by

Byoung S. Ham
Gwangju Institute of Science and Technology, South Korea

A. Theoretical analysis

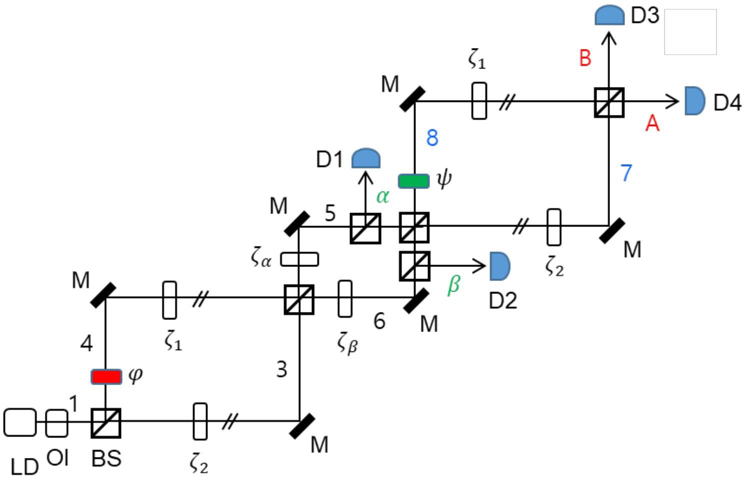

Fig. S1. A schematic of USCKD (see Fig. 1).

In Fig. S1, the matrix representation for the output fields are as follows:

$$\begin{bmatrix} E_A \\ E_B \end{bmatrix} = [MZI]_2[\zeta''] \begin{bmatrix} E_\alpha \\ E_\beta \end{bmatrix},$$

where $\begin{bmatrix} E_\alpha \\ E_\beta \end{bmatrix} = [MZI]_1 \begin{bmatrix} E_1 \\ 0 \end{bmatrix}$, $[MZI]_1 = [BS][\varphi][\zeta][BS]$, $[MZI]_2 = [BS][\psi][\zeta'][BS]$, $[\zeta] = \begin{bmatrix} e^{i\zeta_2} & 0 \\ 0 & e^{i\zeta_1} \end{bmatrix}$, $[\zeta'] = \begin{bmatrix} e^{i\zeta_2'} & 0 \\ 0 & e^{i\zeta_1'} \end{bmatrix}$, $[\zeta''] = \begin{bmatrix} e^{i\zeta_\alpha} & 0 \\ 0 & e^{i\zeta_\beta} \end{bmatrix}$, $[BS] = \frac{1}{\sqrt{2}} \begin{bmatrix} 1 & i \\ i & 1 \end{bmatrix}$, $[\varphi] = \begin{bmatrix} 1 & 0 \\ 0 & e^{i\varphi} \end{bmatrix}$, $[\psi] = \begin{bmatrix} 1 & 0 \\ 0 & e^{i\psi} \end{bmatrix}$, $\zeta = \zeta_2 - \zeta_1$ and $\zeta' = \zeta_2' - \zeta_1'$. In the original scheme of Fig. 1(a), the outbound and inbound traveling light fields propagate through the same paths. Considering the atmospheric turbulence-caused phase noise is less than kHz, the phase difference between the outbound and inbound light fields are negligible if the transmission distance is less than 10 km. For generality, however, the phase noise ζ_j occurred on each transmission channel in Fig. S1 is assumed to be independent.

$$[MZI]_1 = \frac{1}{2} \begin{bmatrix} 1 & i \\ i & 1 \end{bmatrix} \begin{bmatrix} 1 & 0 \\ 0 & e^{i\varphi} \end{bmatrix} \begin{bmatrix} e^{i\zeta_2} & 0 \\ 0 & e^{i\zeta_1} \end{bmatrix} \begin{bmatrix} 1 & i \\ i & 1 \end{bmatrix}$$

$$= \frac{1}{2} e^{i\zeta_1} \begin{bmatrix} 1 & i \\ i & 1 \end{bmatrix} \begin{bmatrix} 1 & 0 \\ 0 & e^{i\varphi} \end{bmatrix} \begin{bmatrix} e^{i\zeta} & 0 \\ 0 & 1 \end{bmatrix} \begin{bmatrix} 1 & i \\ i & 1 \end{bmatrix}$$

$$= \frac{1}{2} e^{i\zeta_1} \begin{bmatrix} 1 & ie^{i\varphi} \\ i & e^{i\varphi} \end{bmatrix} \begin{bmatrix} e^{i\zeta} & ie^{i\zeta} \\ i & 1 \end{bmatrix}$$

$$= \frac{1}{2} e^{i\zeta_1} \begin{bmatrix} e^{i\zeta} - e^{i\varphi} & i(e^{i\zeta} + e^{i\varphi}) \\ i(e^{i\zeta} + e^{i\varphi}) & -(e^{i\zeta} - e^{i\varphi}) \end{bmatrix}$$

$$= \frac{1}{2} e^{i\zeta_2} \begin{bmatrix} 1 - e^{i(\varphi-\zeta)} & i(1 + e^{i(\varphi-\zeta)}) \\ i(1 + e^{i(\varphi-\zeta)}) & -(1 - e^{i(\varphi-\zeta)}) \end{bmatrix}$$

$$= \frac{1}{2} e^{i\zeta_2} \begin{bmatrix} 1 - e^{i\varphi'} & i(1 + e^{i\varphi'}) \\ i(1 + e^{i\varphi'}) & -(1 - e^{i\varphi'}) \end{bmatrix}, \qquad \text{(S1)}$$

where $\varphi' = \varphi - \zeta$.

$$[MZI]_2=\frac{1}{2}\begin{bmatrix}1 & i\\ i & 1\end{bmatrix}\begin{bmatrix}1 & 0\\ 0 & e^{i\psi}\end{bmatrix}\begin{bmatrix}e^{i\zeta_2'} & 0\\ 0 & e^{i\zeta_1'}\end{bmatrix}\begin{bmatrix}1 & i\\ i & 1\end{bmatrix}$$
$$=\frac{1}{2}e^{i\zeta_1'}\begin{bmatrix}1 & i\\ i & 1\end{bmatrix}\begin{bmatrix}1 & 0\\ 0 & e^{i\psi}\end{bmatrix}\begin{bmatrix}e^{i\zeta'} & 0\\ 0 & 1\end{bmatrix}\begin{bmatrix}1 & i\\ i & 1\end{bmatrix}$$
$$=\frac{1}{2}e^{i\zeta_1'}\begin{bmatrix}1 & ie^{i\psi}\\ i & e^{i\psi}\end{bmatrix}\begin{bmatrix}e^{i\zeta'} & ie^{i\zeta'}\\ i & 1\end{bmatrix}$$
$$=\frac{1}{2}e^{i\zeta_1'}\begin{bmatrix}e^{i\zeta'}-e^{i\psi} & i\left(e^{i\zeta'}+e^{i\psi}\right)\\ i\left(e^{i\zeta'}+e^{i\psi}\right) & -\left(e^{i\zeta'}-e^{i\psi}\right)\end{bmatrix}$$
$$=\frac{1}{2}e^{i\zeta_2'}\begin{bmatrix}1-e^{i\psi'} & i\left(1+e^{i\psi'}\right)\\ i\left(1+e^{i\psi'}\right) & -\left(1-e^{i\psi'}\right)\end{bmatrix}, \quad \text{(S2)}$$
where $\psi'=\psi-\zeta'$.

Thus,
$$\begin{bmatrix}E_A\\ E_B\end{bmatrix}=[MZI]_2[\zeta'']\begin{bmatrix}E_\alpha\\ E_\beta\end{bmatrix}$$
$$=\frac{1}{4}e^{i(\zeta_2+\zeta_2')}\begin{bmatrix}1-e^{i\psi'} & i\left(1+e^{i\psi'}\right)\\ i\left(1+e^{i\psi'}\right) & -\left(1-e^{i\psi'}\right)\end{bmatrix}\begin{bmatrix}e^{i\zeta_\alpha} & 0\\ 0 & e^{i\zeta_\beta}\end{bmatrix}\begin{bmatrix}1-e^{i\varphi'} & i\left(1+e^{i\varphi'}\right)\\ i\left(1+e^{i\varphi'}\right) & -\left(1-e^{i\varphi'}\right)\end{bmatrix}\begin{bmatrix}E_0\\ 0\end{bmatrix}$$
$$=\frac{1}{4}e^{i(\zeta_2+\zeta_2'+\zeta_\beta)}\begin{bmatrix}1-e^{i\psi'} & i\left(1+e^{i\psi'}\right)\\ i\left(1+e^{i\psi'}\right) & -\left(1-e^{i\psi'}\right)\end{bmatrix}\begin{bmatrix}e^{i\zeta''} & 0\\ 0 & 1\end{bmatrix}\begin{bmatrix}1-e^{i\varphi'} & i\left(1+e^{i\varphi'}\right)\\ i\left(1+e^{i\varphi'}\right) & -\left(1-e^{i\varphi'}\right)\end{bmatrix}\begin{bmatrix}E_0\\ 0\end{bmatrix}$$
$$=\frac{1}{4}e^{i(\zeta_2+\zeta_2'+\zeta_\beta)}\begin{bmatrix}e^{i\zeta''}\left(1-e^{i\psi'}\right) & i\left(1+e^{i\psi'}\right)\\ ie^{i\zeta''}\left(1+e^{i\psi'}\right) & -\left(1-e^{i\psi'}\right)\end{bmatrix}\begin{bmatrix}1-e^{i\varphi'} & i\left(1+e^{i\varphi'}\right)\\ i\left(1+e^{i\varphi'}\right) & -\left(1-e^{i\varphi'}\right)\end{bmatrix}\begin{bmatrix}E_0\\ 0\end{bmatrix}$$
$$=\frac{1}{4}e^{i(\zeta_2+\zeta_2'+\zeta_\beta)}\cdot$$
$$\begin{bmatrix}e^{i\zeta''}\left(1-e^{i\psi'}\right)\left(1-e^{i\varphi'}\right)-\left(1+e^{i\psi'}\right)\left(1+e^{i\varphi'}\right) & i\left[e^{i\zeta''}\left(1-e^{i\psi'}\right)\left(1+e^{i\varphi'}\right)-\left(1+e^{i\psi'}\right)\left(1-e^{i\varphi'}\right)\right]\\ i\left[e^{i\zeta''}\left(1+e^{i\psi'}\right)\left(1-e^{i\varphi'}\right)-\left(1-e^{i\psi'}\right)\left(1+e^{i\varphi'}\right)\right] & -e^{i\zeta''}\left(1+e^{i\psi'}\right)\left(1+e^{i\varphi'}\right)+\left(1-e^{i\psi'}\right)\left(1-e^{i\varphi'}\right)\end{bmatrix}$$
$$\cdot\begin{bmatrix}E_0\\ 0\end{bmatrix}$$
$$=\frac{1}{4}e^{i(\zeta_2+\zeta_2'+\zeta_\beta)}\cdot$$
$$\begin{bmatrix}-\left(1-e^{i\zeta''}\right)\left(1+e^{i(\varphi'+\psi')}\right)-\left(1+e^{i\zeta''}\right)\left(e^{i\varphi'}+e^{i\psi'}\right) & -i\left[\left(1-e^{i\zeta''}\right)\left(1-e^{i(\varphi'+\psi')}\right)+\left(1+e^{i\zeta''}\right)\left(e^{i\psi'}-e^{i\varphi'}\right)\right]\\ -i\left[\left(1-e^{i\zeta''}\right)\left(1-e^{i(\varphi'+\psi')}\right)+\left(1+e^{i\zeta''}\right)\left(e^{i\psi'}-e^{i\varphi'}\right)\right] & \left(1-e^{i\zeta''}\right)\left(1+e^{i(\varphi'+\psi')}\right)-\left(1+e^{i\zeta''}\right)\left(e^{i\varphi'}+e^{i\psi'}\right)\end{bmatrix}$$
$$\cdot\begin{bmatrix}E_0\\ 0\end{bmatrix} \quad \text{(S3)}$$

B. Numerical calculations for both random phase noises

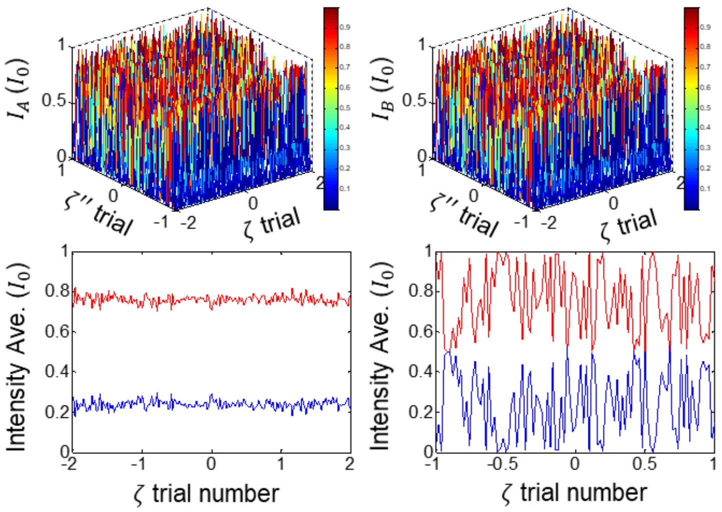

Fig. S2. Numerical calculations of equation (S3) for random phase noises caused by environment. $\varphi \neq \psi$ & $\varphi + \psi = \pi$. The range of random phase noise of both ζ and ζ'' is maximum (2π).

Figure S2 shows the numerical calculations of equation (S3) for $\varphi \neq \psi$ and $\varphi + \psi = \pi$, resulting in inversion relation between input and output in USCKD [30]. The upper panel of Fig. S2 shows full range of noise in the output fields due to random phase fluctuations. As shown in the lower panel, however, the average over the random phase noise with respect to each ζ shows clear separation between two output intensity. The random phase noise effect shown in Fig. S2 is the same as Fig. 2 in the main text.

C. Numerical calculation for linear phase noise of ζ''

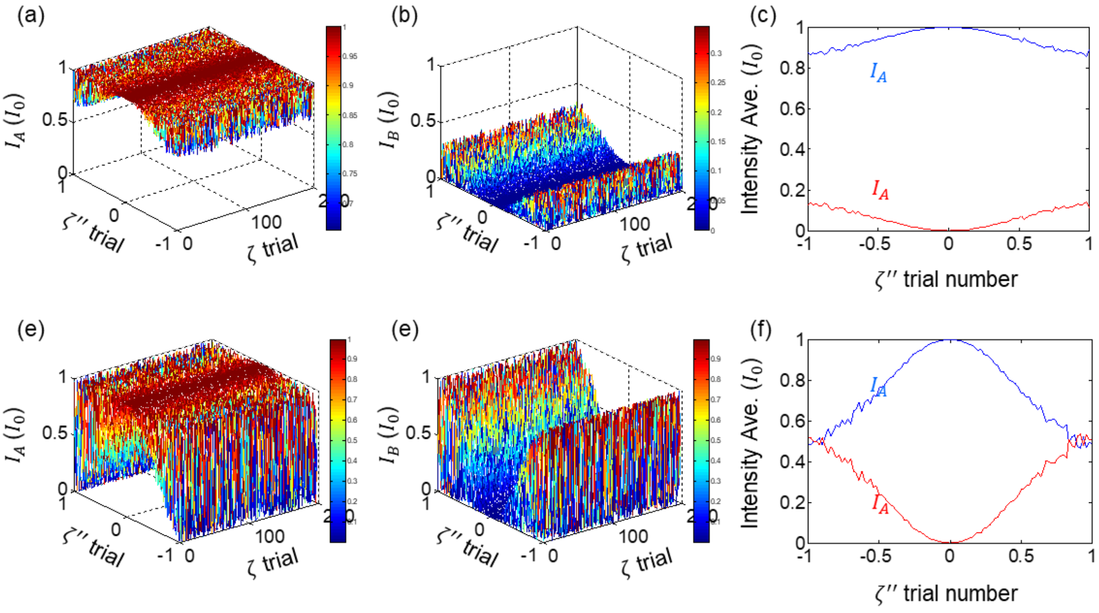

Fig. S3. Numerical calculations of equation (S3) for a linear phase noise of ζ'' with respect to random phase noise range of ζ. (a)-(c) $\zeta = 0.2\pi$. (d)-(f) $\zeta = 2\pi$.

Figure S3 shows the output fields affected by phase noises, where ζ'' range is changed linearly with respect to random noise of ζ. As already shown, there is no intensity fluctuation in both output fields if $\zeta'' = 0$ no matter how big ζ fluctuation is. In Fig. S3(a)-(c), the phase noise range of ζ is $2\pi/10$. As the range of ζ'' increases from 0 to π, the individual output fields are strongly affected by the random phase noise of ζ, but still far less than 50% resulting in complete separation between I_A and I_B. The average over ζ is very stable as shown in Fig. S3(c). For the range of ζ is maximum of π, however, individual output fields fluctuate fully between 0 and maximum at heavy noise of ζ''. Even in this case the average of output fields over ζ is still good with a little crossover at the maximum phase noise of ζ'' of π.

Figure S4 shows numerical calculations of equation (4) for both linearly varying ζ'' and ζ. Both parameters of ζ'' and ζ indicate random phase noise range, e.g., ζ''=0.5 means that the phase ζ'' randomly fluctuates between $0 - 0.5\pi$. More importantly, the program is set for simultaneously varying ζ', ζ, and $\Delta\zeta$, where individual channel noise is not important but relative noise $\Delta\zeta$ plays an important role as driven in equations (6) and (7). From the upper panel of Fig. S4, the importance of $\Delta\zeta$ is represented for USCKD. In other words, the key distribution error through atmospheric turbulence is nearly free if $\Delta\zeta < 0.1$ regardless of ζ''. If ζ'' is also limited in a similar short varying range of $\Delta\zeta$, then the error-free range increases nearly twice up to 0.2. Such a phase noise variation can be maintained without an active control, which is a great benefit for potential applications especially for portable devices.

The lower panels are for details of the upper panels. In the lower left panel, the intensity fluctuation of the output fields are perfect if $\Delta\zeta = 0$ without an error regardless of ζ''. As $\Delta\zeta$ increases, the intensity fluctuation increases and cross over the half line if $\Delta\zeta > 0.4$. However, the output fields' error is reduced if ζ'' is also limited to the similar range to $\Delta\zeta$. The lower right panel shows average outputs with respect to each noise parameter. For the average over ζ'', the intensity fluctuation of I_A is greater than $0.75I_0$ (50% visibility) if $\Delta\zeta < 0.3$. For the average over $\Delta\zeta$, the intensity of I_A lies in the range of $0.5 < I_A < 0.7$ regardless of ζ''. If ζ'' is reduced down to 0.2, then the variation of I_A becomes quite stable at ~$0.7I_0$ (40% visibility). In any cases, the average effect of each intensity indicates a good maintenance from the other keeping its intensity level higher (lower to I_A; not shown) than the half value. Thus, the present USCKD scheme is robust to the environmental phase noise.

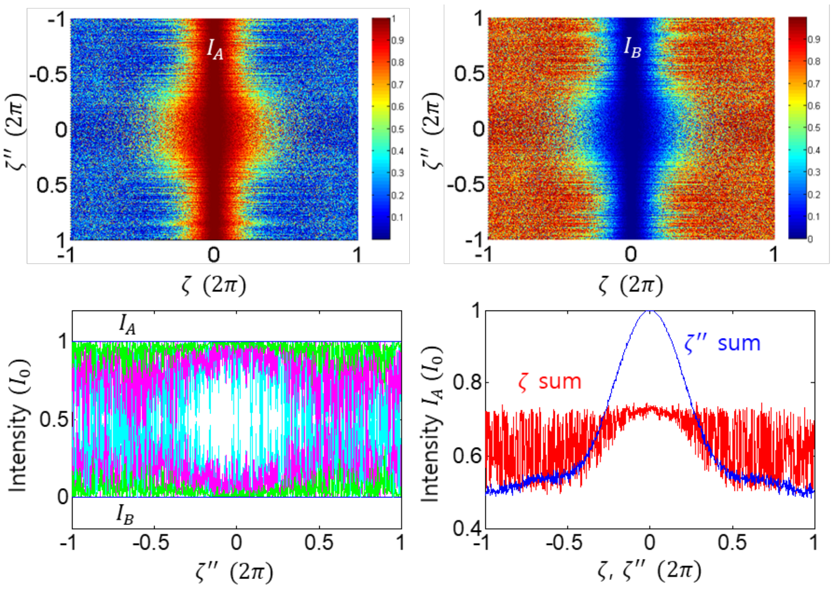

Fig. S4. Numerical calculations for environment noise caused output field intensity fluctuations. In the lower left panel: $\zeta = 0$(blue, 0 & 1); 0.2π(green); 0.4π(magenta); 0.6π(cyan). $\zeta' = \zeta = \Delta\zeta$.
